# Effect of maternal obesity with and without gestational diabetes on offspring subcutaneous and preperitoneal adipose tissue development from birth up to year-1

**DOI:** 10.1186/1471-2393-14-138

**Published:** 2014-04-11

**Authors:** Kirsten Uebel, Karina Pusch, Kurt Gedrich, Karl-Theo M Schneider, Hans Hauner, Bernhard L Bader

**Affiliations:** 1 PhD Graduate School ‘Nutritional adaptation and epigenetic mechanisms’, ZIEL - Research Center for Nutrition and Food Sciences, Technische Universität München, Freising-Weihenstephan, Germany; 2 Clinical Nutritional Medicine Unit, ZIEL - Research Center for Nutrition and Food Sciences, Technische Universität München, Freising-Weihenstephan, Germany; 3 Biochemistry Unit, ZIEL - Research Center for Nutrition and Food Sciences, Technische Universität München, Freising-Weihenstephan, Germany; 4 Else Kröner-Fresenius-Center for Nutritional Medicine, Wissenschaftszentrum Weihenstephan, Klinikum rechts der Isar, Uptown München Campus D, Technische Universität München, Munich, Germany; 5 Abteilung für Geburtshilfe und Perinatalmedizin der Frauenklinik, Technische Universität München, Munich, Germany

**Keywords:** Gestational diabetes mellitus, Maternal obesity, Fetal programming, Adiponectin, Insulin, C-peptide, Skinfold thickness, Ultrasonography, Subcutaneous adipose tissue, Preperitoneal adipose tissue

## Abstract

**Background:**

Maternal obesity and gestational diabetes mellitus (GDM) may independently influence offspring fat mass and metabolic disease susceptibility. In this pilot study, body composition and fat distribution in offspring from obese women with and without GDM and lean women were assessed within the 1st year of life, and maternal and newborn plasma factors were related to offspring adipose tissue distribution.

**Methods:**

Serum and plasma samples from pregnant obese women with (n = 16) or without (n = 13) GDM and normoglycemic lean women (n = 15) at 3rd trimester and offspring cord plasma were used for analyzing lipid profiles, insulin and adipokine levels. At week-1 and 6, month-4 and year-1, offspring anthropometrics and skinfold thickness (SFT) were measured and abdominal subcutaneous (SCA) and preperitoneal adipose tissue (PPA) were determined by ultrasonography.

**Results:**

Cord insulin was significantly increased in the GDM group, whereas levels of cord leptin, total and high molecular weight (HMW) adiponectin were similar between the groups. Neonates of the GDM group showed significantly higher SFT and fat mass until week-6 and significantly increased SCA at week-1 compared to the lean group that persisted as strong trend at week-6. Interestingly, PPA in neonates of the GDM group was significantly elevated at week-1 compared to both the lean and obese group. At month-4 and year-1, significant differences in adipose tissue growth between the groups were not observed. Multiple linear regression analyses revealed that cord insulin levels are independently related to neonatal PPA that showed significant relation to PPA development at year-1. Maternal fasted C-peptide and HMW adiponectin levels at 3rd trimester emerged to be determinants for PPA at week-1.

**Conclusion:**

Maternal pregravid obesity combined with GDM leads to newborn hyperinsulinemia and increased offspring fat mass until week-6, whereas pregravid obesity without GDM does not. This strongly suggests the pivotal role of GDM in the adverse offspring outcome. Maternal C-peptide and HMW adiponectin levels in pregnancy emerge to be predictive for elevated PPA in newborns and might be indicative for the obesity risk at later life. Altogether, the findings from our pilot study warrant evaluation in long-term studies.

**Trial registration:**

German Clinical Trials Register DRKS00004370

## Background

Emerging data suggest a permanent impact of the perinatal environment on the offspring risk for developing obesity, metabolic and cardiovascular diseases. The prevalence of obesity in US children and adolescents was 16.9% in 2009–2010 [1], while a German survey showed slightly lower rates [2]. Particularly worrying is the continuous increase in obesity prevalence within young women showing that more than 20.5% of US women at reproductive age were obese (BMI > 30 kg/m^2^) in 2009 [3]. A similar trend was recently reported for the German population [4].

Pregravid obesity is, besides maternal age, ethnicity and family history of diabetes, also a major predictor for gestational diabetes mellitus (GDM) [5]. Importantly, maternal obesity and GDM are independent risk factors for fetal hyperinsulinemia, elevated weight and body fat at birth, suggesting that both GDM and pregravid obesity influence fetal development through similar mechanism [6-8]. Moreover, children born from obese and/or diabetic mothers are at higher risk for obesity and insulin resistance/type 2 diabetes from childhood periods [9-11] and adolescence [11,12] up to adulthood [13,14].

There is rising evidence from infants and adults that the distribution of fat depots is a stronger predictor for the metabolic and cardiovascular risk than the total amount of fat [15,16]. Notably, visceral fat has been associated with insulin resistance, decreased adiponectin and hypertriglyceridemia in children and adolescents [17]. Recently, ultrasonography was considered as a valid method for measuring infant abdominal fat distribution in clinical research [18,19]. Preperitoneal adipose tissue (PPA) assessed by ultrasonography has been proposed as a surrogate for visceral/intra-abdominal fat. However, there is missing information about metabolic markers, e.g. insulin, leptin and adiponectin, associated with the distribution of abdominal subcutaneous (SCA) adipose tissues and PPA in neonates and early infancy.

This pilot study comprised pregravid obese women with and without GDM and lean pregnant women, as well as their offspring. Maternal and umbilical cord plasma metabolic markers were determined and related to offspring anthropometrics and distribution of adipose tissues, skinfold thickness (SFT) and abdominal SCA and PPA, from birth/week-1 up to year-1 *post partum (pp)*.

## Methods

### Study population

The study protocol of the *GesA*-Study (**Ges**tational diabetes mellitus and **a**diposity in early adipose tissue development) was approved by the Ethics Committee of the Technische Universität München (Ethics Committee Number: 2629/09) and was registered at the German clinical trials register (DRKS00004370). Singleton pregnant women were recruited at the Abteilung für Geburtshilfe und Perinatalmedizin der Frauenklinik, Klinikum rechts der Isar, Technische Universität München, Munich, Germany between week-33 and 36 of gestation. Written informed consent was obtained from participants before their first appointment. Exclusion criteria for pregnant women were preeclampsia, chronic hypertension and pregravid type 1 or type 2 diabetes. At delivery, participants were excluded if newborns were born preterm (< 37 weeks of gestation), small for gestational age (SGA) or with an APGAR < 5. The study group consisted of Caucasian pregnant women divided into three groups according to their self-reported pregravid BMI and the result of the oral glucose tolerance tests in the 2nd and 3rd trimester [normoglycemic lean (BMI 18.5-25 kg/m^2^; n = 15), obese women without GDM (BMI > 30 kg/m^2^; n = 13), and obese women with GDM (BMI > 30 kg/m^2^; diet-controlled n = 7, insulin-treated n = 9)]. The diagnosis of GDM was defined according to the Hyperglycemia and Pregnancy Outcome (HAPO) criteria [20]: fasting glucose > 5.1 mmol/L (92 mg/dL), 1 h glucose > 10 mmol/L (180 mg/dL), 2 h glucose > 8.5 mmol/L (153 mg/dL). GDM was diagnosed if at least 1 of the threshholds was met or exceeded. Women, tested negatively in the OGTT in 2nd trimester, were retested in the 3rd trimester to confirm glucose tolerance and to re-evaluate their group allocation.

### Biochemical analysis

Venous blood samples were obtained from fasted women between week-33 and 36 of gestation. Neonatal blood was collected immediately after delivery from umbilical cord vein. At Synlab (Munich, Germany), maternal and cord plasma glucose were assessed by the glucose oxidase method, and maternal and fetal serum lipid parameters (total, LDL and HDL cholesterol, triglycerides) were determined using established enzymatic methods (Roche Diagnostics, Mannheim, Germany). Fasted maternal plasma and cord plasma insulin as well as fasted maternal plasma C-peptide were analyzed using commercially available ELISAs (Dako, Glostrup, Denmark). Maternal insulin sensitivity was assessed according to the formula for the homeostasis model of assessment-insulin resistance: HOMA-IR-index = [Insulin (μU/mL) × Glucose (mmol/L)]/22.5. Maternal and cord plasma levels for leptin (R&D Systems, Minneapolis, USA), total adiponectin and HMW adiponectin (ALPCO, Salem, NH, USA) were determined by ELISA. The ratio of high molecular weight (HMW) to total adiponectin (S_A_) was calculated for each plasma sample. All calculated intra- and interassay coefficients of variation (CV) were less than 10%.

### Measurements of anthropometric parameters and adipose tissue distribution

Anthropometric data and SFT were assessed as originally described by Hauner et al. [21]. Briefly, birth weight, height and head circumference were obtained from the obstetric protocol. At visit week-6, month-4 and year-1 *pp*, the infant weight and height, head circumference, and waist circumference (only at year-1) were determined in the study center. Ponderal index (in kg/m^3^) was calculated according to the measured variables.

SFT measurements were conducted by one well-trained researcher at 2–5 days *pp* in the obstetric clinic as well as at week-6, month-4 and year-1 *pp* at the study center. SFTs were determined in triplicate with a Holtain caliper (Holtain Ltd, Crosswell, Crymych, UK) at the infant’s left body axis at the following four sites: triceps, biceps, subscapular and suprailiac. Total body fat was calculated via predictive SFT equation according to the method of Westrate and Deurenberg [22] and trunk-to total SFT (%) was evaluated by applying the equation (subscapular + suprailiac SFT)/(sum of 4 SFTs) × 100 [23].

Ultrasonographic imaging, as described originally by Holzhauer et al. [18] was performed by two researchers (including one pediatrician blinded to study group allocation) at week-1, week-6, month-4 and year-1. Briefly, abdominal SCA and PPA fat thicknesses were measured in sagittal planes as areas of 1-cm length just below the xiphoid process. Additionally, SCA was determined in axial planes as areas of 1-cm length in the middle of the xiphoid process and the navel directly above the linea alba. From each plane, three images were analyzed by one observer and 120 images were randomly controlled by a completely blinded second researcher. The inter-observer variability was calculated by interclass coefficient (ICC) and further validated by a Bland-Altman plot [24]. The ICC for the ultrasonographic analysis was 0.981 and the p-value < 0.001, indicating high correlation between independent assessments. In the Bland-Altman plot analysis, the mean of the differences (bias) between observers was −0.17 mm^2^ and the *Limits of Agreement* were −0.65 mm^2^ (mean −2 SD) and 0.31 mm^2^ (mean +2 SD), excluding a systematic inter-observer bias (Additional file 1).

### Statistical analysis

For all statistical analyses, IBM SPSS statistics software (version 20.0; IBM Corp.) was used. One-way ANOVA with Sidak’s post hoc test was applied for group comparison of normally distributed variables and data are presented as means ± SD. In case of violation of the normality assumption, the Kruskal-Wallis test with Dunn’s post hoc test was applied, and accordingly, data are presented as medians and interquartile ranges. The Fisher’s Exact test was used for analyzing qualitative measurements. Comparative statistical evaluation of infant growth and fat development between the groups was performed by employing multiple linear regression models. The potential confounding factors offspring sex and pregnancy duration were taken into account when analyzing cord parameters and the covariate infant age at investigation was additionally applied for respective anthropometric and adipose tissue growth assessments. Generally, infant parameters at week-6, month-4 and year-1 were further adjusted for breastfeeding status.

Linear regression analyses were used to identify maternal parameters and cord insulin levels independently related to offspring adipose tissue distribution. If necessary, logarithmically transformed variables were applied to accomplish normality of residuals. Values for the unadjusted analyses are presented, whereas the indicated adjusted model considered the variables offspring sex, gestational duration, maternal pre-pregnancy BMI, area under the curve (AUC) _Glucose_ (OGTT) and gestational weight gain. Applying adipose tissue markers at week-6, month-4 and year-1 as dependent variables, corresponding breastfeeding status was a further covariate. Spearman correlation analysis was used to determine the association of cord adiponectin levels with respective cord insulin levels and adipose tissue parameters at week-1, and respective partial correlations were applied including the covariables for infant sex, pregnancy duration, maternal pre-pregnancy BMI, AUC _Glucose_ (OGTT) and gestational weight gain. All statistical tests were performed two-sided and a p-value < 0.05 was considered to indicate statistical significance.

## Results

### Maternal and fetal metabolic outcome

The main clinical and metabolic data of the study population are summarized in Table 1. Lean and obese participants with and without GDM did not significantly differ in parity or mode of delivery, but obese participants with GDM were slightly older at study entry, and pregnancy duration was shorter compared to obese subjects without GDM. Both obese groups had similar pregravid BMI and weight gain during pregnancy, whereas the glucose levels (fasted, 1 h, 2 h) upon OGTT were significantly higher in the obese GDM group than in the obese group without GDM. At baseline (week-33 to 36 of gestation), obese women with and without GDM had significantly increased levels of fasting insulin and C-peptide compared to lean subjects and showed increased insulin resistance estimated by HOMA-IR-index. HbA1c levels were similar between all groups. Plasma leptin levels of obese pregnant women were - irrespective of GDM - significantly higher, while HMW-total adiponectin ratios (S_A_) were significantly lower compared to the lean group. Concerning the lipid profile, total cholesterol levels were significantly decreased in obese subjects, but HDL cholesterol and triglyceride levels were similar between study groups. Cord plasma insulin levels and HOMA-IR-index were significantly elevated in the obese group with GDM compared to the euglycemic obese and lean group. In contrast, no significant differences were observed for cord plasma HMW and total adiponectin or S_A_ between all study groups. In addition, levels of LDL, HDL, and total cholesterol as well as triglycerides were similar in all groups. With regard to breastfeeding status at week-6 and month-4 and the introduction of solid food, no significant differences were observed between the study groups.

**Table 1:** Characterization of study participants

**A Mother**	**Lean group**	**Obese group**	**Obese GDM group**	**P-value**^**1**^	
Number of women (n = 44)	15	13	16		
Age (year)	31.1 ± 3.1	28.5 ± 4.2	32.6 ± 4.8	0.040^c^	
Primiparae (%) [n]	86.7 [13]	76.9 [10]	56.2 [9]	0.299	
BMI (kg/m^2^) before pregnancy	20.1 (19.5-22.0)	36.1 (32.2-38.3)	33.4 (31.1-36.1)	< 0.001^a,b^	
Gestational weight gain (kg)	16.8 ± 4.4	11.6 ± 5.6	12.1 ± 5.8	0.022^a,b^	
Parental history of diabetes (%) [n]	13.3 [2]	38.5 [5]	43.8 [7]	0.181	
Duration of pregnancy (days)	278.2 ± 8.1	279.5 ± 8.2	271.3 ± 7.0	0.012^c^	
**Mode of delivery**					
Spontaneous / cesarean section, n	9/6	6/7	6/10	0.483	
**75 g OGTT (mmol/L)**					
Glucose fasted	4.22 (3.89-4.39)	4.33 (4.00-4.63)	5.11 (4.44-5.56)	< 0.001^b,c^	
Glucose 1 h	7.41 ± 0.90	7.34 ± 1.42	10.85 ± 1.34	< 0.001^b,c^	
Glucose 2 h	6.30 ± 0.96	6.49 ± 1.21	8.00 ± 1.57	< 0.001^b,c^	
Area under curve (AUC) _Glucose_	12.54 (11.90-13.24)	11.85 (11.65-14.10)	16.37 (16.04-18.13)	< 0.001^b,c^	
**Fasted plasma/serum parameters at 3rd trimester**					
Gestational age (weeks)	33.9 ± 1.4	34.3 ± 1.7	34.1 ± 1.0	0.836	
Glucose (mmol/L)	4.10 ± 0.32	4.19 ± 0.40	4.39 ± 0.28	0.161	
Insulin (μU/mL)	5.66 (4.76-10.05)	11.54 (9.28-20.54)	13.99 (11.22-17.97)	0.002^a,b^	
HOMA-IR-index	1.10 (0.86-1.79)	2.31 (1.98-4.15)	2.76 (2.08-3.63)	0.001^a,b^	
C-peptide (ng/mL)	1.60 (1.42-2.23)	2.99 (2.05-3.51)	3.01 (2.64-3.14)	0.002^a,b^	
HbA1c (%)	5.32 ± 0.31	5.45 ± 0.23	5.40 ± 0.28	0.474	
Leptin (ng/mL)	9.30 (6.52-20.11)	46.03 (29.84-78.67)	42.24 (26.71-66.69)	< 0.001^a,b^	
Total adiponectin (μg/mL)	4.98 ± 1.02	4.14 ± 1.78	3.81 ± 1.39	0.071	
HMW adiponectin (μg/mL)	2.86 ± 0.82	2.06 ± 1.17	1.74 ± 0.91	0.008^b^	
S_A_	0.57 ± 0.1	0.48 ± 0.09	0.44 ± 0.07	< 0.001^a,b^	
Total cholesterol (mmol/L)	7.49 ± 1.51	6.28 ± 0.96	6.60 ± 0.96	0.035^a^	
LDL cholesterol (mmol/L)	4.86 ± 1.50	3.73 ± 0.87	3.92 ± 1.96	0.039	
HDL cholesterol (mmol/L)	1.92 ± 0.49	1.73 ± 0.45	1.88 ± 0.28	0.524	
Triglycerides (mmol/L)	2.53 ± 0.74	2.38 ± 0.57	2.76 ± 0.72	0.420	
**B Infant**	**Lean group**	**Obese group**	**Obese GDM group**	**P-value**^**1**^	**Adjusted p-value**^**2**^
Offspring gender (male/female), n	8/7	8/5	13/3	0.275	
**Umbilical cord plasma parameters**					
Glucose (mmol/L)	4.46 ± 1.13	4.42 ± 1.03	4.60 ± 1.02	0.892	0.202
Insulin (μU/mL)	1.72 (1.08-5.14)	1.73 (1.33-3.97)	5.29 (4.21-8.33)	0.023^b,c^	0.014^b,c^
HOMA-IR-index	0.40 (0.22-1.23)	0.42 (0.23-0.74)	1.08 (0.81-1.52)	0.021^b,c^	0.009^b,c^
Leptin (ng/mL)	5.88 (2.15-9.70)	7.57 (4.56-16.65)	6.84 (4.76-11.58)	0.455	0.114
Total adiponectin (μg/mL)	20.68 ± 6.23	20.82 ± 8.56	22.03 ± 7.33	0.977	0.685
HMW adiponectin (μg/mL)	14.61 ± 5.15	14.28 ± 7.02	14.75 ± 5.80	0.860	0.884
S_A_	0.70 ± 0.06	0.67 ± 0.06	0.65 ± 0.08	0.292	0.348
Total cholesterol (mmol/L)	1.71 (1.31-1.90)	1.53 (1.36-1.71)	1.55 (1.32-1.73)	0.718	0.886
LDL cholesterol (mmol/L)	0.54 (0.32-0.65)	0.52 (0.38-0.60)	0.47 (0.41-0.54)	0.905	0.271
HDL cholesterol (mmol/L)	0.72 (0.67-1.03)	0.62 (0.60-0.96)	0.72 (0.57-0.88)	0.691	0.724
Triglycerides (mmol/L)	0.35 (0.27-0.52)	0.35 (0.26-0.45)	0.40 (0.25-0.46)	0.959	0.847
**Breastfeeding status at week-6 (month-4), n**					
Exclusively breastfed	10 (8)	7 (6)	6 (4)	0.257 (0.304)	
Formula and breast milk	0 (2)	3 (0)	4 (4)		
Formula only	5 (5)	3 (6)	4 (6)		
Introduction of solid food (months)	5.0 (5.0-6.0)	5.0 (4.0-6.0)	5.0 (4.0-6.0)	0.411	

HbA1c: hemoglobin A1c; HDL: high-density lipoprotein; HOMA-IR: homeostasis model assessment-insulin resistance; LDL: low-density lipoprotein; OGTT: oral glucose tolerance test; S_A_: HMW-total adiponectin ratio.

Data are presented as mean ± SD; otherwise skewed variables are presented as median followed by interquartile range.

^1^One-way ANOVA with Sidak’s post hoc test or nonparametric Kruskal-Wallis-test with Dunn’s post hoc test.

^2^Statistical analyses for cord plasma parameters were performed by multiple linear regression models and p-values were adjusted for infant sex and pregnancy duration.

^a^P-value < 0.05 obese vs. lean group; ^b^p-value < 0.05 obese GDM vs. lean group; ^c^p-value < 0.05 obese GDM vs. obese group.

### Infant anthropometrics, adipose tissue growth and distribution

Infant anthropometrics and adipose tissue development assessed by SFT measurement and ultrasonography from birth/week-1 to year-1 are shown in Tables 2 and 3, respectively. Anthropometric birth outcomes, namely weight, height, ponderal index and head circumference were similar for all groups. However, the sum of 4 SFT at week-1 was significantly higher in the obese GDM group than in the lean group after considering the potential confounders infant sex, pregnancy duration and age at investigation. Similarly, abdominal SCA was significantly elevated in the newborns of obese mothers with GDM compared to the lean group, whereas PPA was significantly increased compared to the normoglycemic lean and obese group. At week-6, only SFT and calculated fat mass were significantly higher in the obese group with GDM after considering indicated confounders. In contrast, SCA and PPA were not significantly larger in offspring of obese mothers with GDM at week-6. Notably, although the sum of all 4 SFT did not differ significantly between groups at year-1, the trunk-total SFT ratio was significantly higher in obese GDM offspring than in children of both euglycemic groups, indicating changes in adipose tissue distribution with increased trunk fat in these infants. With regard to further anthropometric and adipose tissue parameters at month-4 and year-1 and total weight and fat mass gain from birth to year-1, no significant differences were observed between the three groups (Tables 2 and 3).

**Table 2:** Anthropometric infant data from birth to year-1

	**Lean group**	**Obese group**	**Obese GDM group**	**P-value**^**1**^	**Adjusted p-value**^**2**^
**Weight [g]**					
Birth	3,449 ± 332 [15]	3,651 ± 285 [13]	3,617 ± 333 [16]	0.201	0.169
Week-6	4,701 ± 330 [15]	4,963 ± 635 [13]	5,285 ± 517 [12]	0.017^b^	0.170
Month-4	6,468 ± 522 [15]	6,929 ± 829 [11]	7,066 ± 732 [13]	0.071	0.211
Year-1	9,786 ± 915 [15]	10,356 ± 1273 [12]	10,392 ± 1212 [14]	0.286	0.549
**Weight gain [g]**					
Δ Week-6 - birth	1,265 (1,095-1,480) [15]	1,480 (775–1,690) [13]	1,550 (1,361-1,892) [12]	0.017^b^	0.394
Δ Month-4 - week-6	1,734 (1,390-2,100) [15]	2,070 (1,840-2,300) [11]	1,848 (1,460-2,100) [11]	0.112	0.042^a^
Δ Year-1 - month-4	3,318 ± 760 [15]	3,228 ± 801 [11]	3,329 ± 666 [13]	0.936	0.937
Δ Year-1 - birth	6,337 ± 996 [15]	6,665 ± 1,220 [12]	6,765 ± 1,102 [14]	0.554	0.722
**Length [cm]**					
Birth	51.8 ± 1.5 [15]	52.5 ± 1.4 [13]	52.3 ± 1.5 [16]	0.299	0.264
Week-6	55.6 ± 0.9 [15]	56.0 ± 1.8 [13]	56.4 ± 1.4 [12]	0.360	0.738
Month-4	63.3 ± 1.2 [15]	64.4 ± 2.0 [11]	63.7 ± 1.4 [13]	0.226	0.177
Year-1	76.5 ± 2.3 [15]	77.5 ± 2.6 [12]	76.7 ± 2.7 [14]	0.551	0.666
**Ponderal index [g/cm**^***3***^**]**					
Birth	6.65 ± 0.54 [15]	6.95 ± 0.48 [13]	6.81 ± 0.51 [16]	0.291	0.287
Week-6	8.45 ± 0.55 [15]	8.86 ± 1.03 [13]	9.36 ± 0.75 [12]	0.018^b^	0.176
Month-4	10.21 ± 0.81 [15]	10.74 ± 1.06 [11]	11.04 ± 0.97 [13]	0.073	0.272
Year-1	12.80 ± 1.04 [15]	13.34 ± 1.38 [12]	13.52 ± 1.30 [14]	0.262	0.616
**Head circumference [cm]**					
Birth	34.0 (34.0-35.5) [15]	35.0 (34.5-36.5) [13]	35.5(34.0-36.0) [16]	0.072	0.410
Week-6	37.5 (37.0-38.8) [15]	38.3 (37.6-39.7) [13]	38.9 (37.7-40.0) [12]	0.023^b^	0.096
Month-4	41.1 ± 1.2 [15]	42.1 ± 1.7 [11]	42.0 ± 1.3 [13]	0.132	0.184
Year-1	45.6 ± 1.4 [15]	47.0 ± 1.6 [12]	47.1 ± 1.7 [14]	0.072	0.105
**Waist circumference [cm]**					
Year-1	45.1 ± 2.4 [15]	45.6 ± 4.0 [12]	45.7 ± 3.0 [14]	0.859	0.897

Data are presented as mean ± SD; otherwise skewed variables are presented as median followed by interquartile range, numbers in brackets represent number of infants included in the analysis.

^1^One-way ANOVA with Sidak’s post hoc test or nonparametric Kruskal-Wallis-test with Dunn’s post hoc test.

^2^Statistical analyses were performed by multiple linear regression models. P-values for parameters at week-1 were adjusted for infant sex, pregnancy duration and age at investigation. P-values at week-6, month-4 and year-1 were adjusted for infant sex, pregnancy duration, age at investigation and corresponding breastfeeding.

^a^P-value < 0.05 obese vs. lean group; ^b^p-value < 0.05 obese GDM vs. lean group.

**Table 3:** Infant adipose tissue growth assessed by skinfold thickness measurements and ultrasonography from week-1 to year-1

	**Lean group**	**Obese group**	**Obese GDM group**	**P-value**^**1**^	**Adjusted p-value**^**2**^
**SFT [mm]**					
Week-1	18.9 ± 3.1 [15]	20.3 ± 2.6 [13]	21.6 ± 2.4 [16]	0.031^a^	0.031^a^
Week-6	25.2 ± 3.7 [15]	26.1 ± 5.1 [13]	30.4 ± 3.4 [12]	0.007^a,b^	0.048^a^
Month-4	30.7 ± 6.6 [15]	31.2 ± 4.3 [11]	33.6 ± 4.8 [13]	0.336	0.258
Year-1	29.0 ± 4.3 [15]	30.3 ± 4.6 [12]	29.7 ± 4.8 [14]	0.749	0.536
**Fat mass [g]**^***3***^					
Week-1	583 ± 139 [15]	660 ± 114 [13]	694 ± 117 [16]	0.051	0.042^a^
Week-6	1,008 ± 159 [15]	1,098 ± 285 [13]	1,307 ± 187 [12]	0.004^a,b^	0.048^a^
Month-4	1,581 ± 318 [15]	1,728 ± 327 [11]	1,847 ± 328 [13]	0.108	0.184
Year-1	2,260 ± 419 [15]	2,475 ± 536 [12]	2,444 ± 530 [14]	0.466	0.498
**Trunk-total SFT ratio (%)**^**4**^					
Week-1	47.4 ± 2.5 [15]	47.2 ± 3.3 [13]	48.1 ± 2.9 [16]	0.630	0.958
Week-6	48.4 ± 3.2 [15]	49.4 ± 3.4 [13]	49.1 ± 2.4 [12]	0.648	0.528
Month-4	49.0 ± 4.1 [15]	48.9 ± 2.4 [11]	49.6 ± 3.1 [13]	0.841	0.647
Year-1	45.1 ± 2.6 [15]	44.9 ± 2.6 [12]	48.5 ± 3.3 [14]	0.002^a,b^	0.035^a,b^
**SCA (mm**^**2**^**)**^**5**^					
Week-1	12.7 (9.5-15.4) [12]	16.7 (14.0-19.9) [12]	17.7 (16.8-19.4) [12]	0.020^a^	0.014^a^
Week-6	24.0 ± 6.6 [15]	26.5 ± 10.7 [13]	34.6 ± 7.9 [12]	0.008^a,b^	0.058^a^
Month-4	41.3 ± 12.8 [15]	42.9 ± 9.4 [11]	45.6 ± 12.7 [13]	0.644	0.458
Year-1	24.6 (18.6-27.7) [15]	29.0 (26.8-36.2) [12]	34.2 (22.8-43.6) [14]	0.132	0.217
**PPA (mm**^**2**^**)**^**5**^					
Week-1	5.6 (4.9-7.9) [12]	7.9 (6.3-9.3) [12]	10.0 (8.9-12.2) [12]	0.001^a^	0.001^a,b^
Week-6	10.0 ± 2.7 [15]	11.4 ± 3.6 [13]	13.9 ± 2.9 [12]	0.009^a^	0.183
Month-4	13.5 (9.7-14.1) [15]	16.9 (12.1-18.6) [11]	15.6 (11.5-18.0) [13]	0.221	0.525
Year-1	17.5 ± 4.9 [15]	22.3 ± 7.0 [12]	20.8 ± 6.7 [14]	0.130	0.368

PPA: preperitoneal adipose tissue; SCA: subcutaneous adipose tissue; SFT: sum of the 4 skinfold thickness measurements (biceps + triceps + subscapular + suprailiac).

Data are presented as mean ± SD; otherwise skewed variables are presented as median followed by interquartile range; numbers in brackets represent number of infants included in the analysis.

^1^One-way ANOVA with Sidak’s post hoc test or nonparametric Kruskal-Wallis-test with Dunn’s post hoc test.

^2^Statistical analyses were performed by multiple linear regression models. P-values for parameters at week-1 were adjusted for infant sex, pregnancy duration and age at investigation. P-values at week-6, month-4 and year-1 were adjusted for infant sex, pregnancy duration, age at investigation and corresponding breastfeeding status.

^a^P-value < 0.05 obese GDM vs. lean group; ^b^p-value < 0.05 obese GDM vs. obese group.

^3^Fat mass was estimated from the sum of 4 SFTs by using the equations of Weststrate and Deurenberg [22].

^4^Trunk-total SFT ratio (%) was evaluated by applying the equation (subscapular + suprailiac SFT)/(sum of 4 SFTs) × 100 of Westrate et al. [23].

^5^SCA and PPA were measured as areas of 1-cm length according to the method of Holzhauer et al. [18]. SCA indicates mean area from axial + sagittal plane.

### Relation of cord plasma insulin to offspring fat distribution up to year-1

Linear regression analyses were used to explore the relation of cord plasma insulin to infant SFT, SCA and PPA at all ages investigated (Table 4). Thus, cord insulin levels were positively related to SFT at week-1 in the unadjusted analysis, however, in the adjusted model, the relationship did not remain significant (p = 0.071). After repeating the linear regressions with abdominal SCA and PPA at week-1, respectively, cord insulin levels also emerged as positive determinant for both adipose tissue markers in the unadjusted analysis (Table 4). This independent relationship still remained after adjusting for infant sex, pregnancy duration, maternal pre-pregnancy BMI, AUC _Glucose_ (OGTT) and gestational weight gain. No significant relationships were found between cord insulin levels and fat distribution at week-6, month-4 and year-1 in the fully adjusted analyses (Table 4).

**Table 4:** Regression of fat distribution parameters up to year-1 on cord plasma insulin levels

**Cord plasma insulin**						
		**Unadjusted analysis**		**Adjusted analysis**		
	**N**	**β**	**P-value**	**β**	**P-value**	**Adjusted model r**^**2**^
**Week-1**						
SFT	44	0.398	0.007	0.325	0.071	0.195; p = 0.026
SCA	36	0.464	0.004	0.390	0.039	0.310; p = 0.008
PPA	36	0.445	0.007	0.436	0.022	0.311; p = 0.008
**Week-6**						
SFT	40	0.294	0.065	0.292	0.146	0.122; p = 0.127
SCA	40	0.335	0.035	0.241	0.217	0.163; p = 0.074
PPA	40	0.307	0.054	0.287	0.119	0.268; p = 0.014
**Month-4**						
SFT	39	0.281	0.084	0.196	0.335	0.080; p = 0.215
SCA	39	0.175	0.287	0.056	0.794	−0.044; p = 0.615
PPA	39	0.146	0.368	0.011	0.961	−0.090; p = 0.799
**Year-1**						
SFT	41	−0.072	0.653	−0.017	0.937	−0.080; p = 0.769
SCA	41	0.027	0.866	0.085	0.680	−0.034; p = 0.585
PPA	41	0.062	0.698	0.198	0.290	0.137; p = 0.099

β: standardized regression coefficient; PPA: preperitoneal adipose tissue; r^2^: coefficient of determination; SCA: subcutaneous adipose tissue; SFT: sum of the 4 skinfold thickness measurements (biceps + triceps + subscapular + suprailiac). Variables for adjusted analysis: infant sex, pregnancy duration, respective breastfeeding status, maternal pre-pregnancy BMI, AUC _Glucose_ (OGTT) and gestational weight gain.

### Relation of infant anthropometric parameters at birth/week-1 to respective year-1 measures

The influence of neonatal anthropometric parameters on corresponding primary endpoint parameters at year-1 were evaluated by linear regression. The adjusted analysis was further performed with the covariates infant sex, pregnancy duration, breastfeeding status at month-4, pre-pregnancy BMI, AUC _Glucose_ (OGTT) and gestational weight gain (Table 5). Values for PPA at week-1 emerged as positive independent determinants of PPA at year-1 in both analyses. In contrast, no further significant relationships were observed between birth weight, ponderal index, SFT and SCA assessed at year-1 and respective parameters determined at birth/week-1.

**Table 5:** Regression of growth and fat mass parameters at year-1 on respective neonatal assessments

**Respective parameters at year-1**						
		**Unadjusted analysis**		**Adjusted analysis**		
	**N**	**β**	**P-value**	**β**	**P-value**	**Adjusted model r**^**2**^
Birth weight	41	0.294	0.062	0.179	0.289	0.149; p = 0.085
Birth ponderal index	41	0.248	0.117	0.161	0.342	0.130; p = 0.109
SFT, week-1	41	−0.012	0.939	−0.082	0.666	−0.074; p = 0.746
SCA, week-1	36	0.006	0.863	−0.260	0.227	−0.260; p = 0.277
PPA, week-1	36	0.520	0.002	0.486	0.010	0.275; p = 0.027

β: standardized regression coefficient; PPA: preperitoneal adipose tissue; r^2^: coefficient of determination; SCA: subcutaneous adipose tissue; SFT: sum of the 4 skinfold thickness measurements (biceps + triceps + subscapular + suprailiac). Variables for adjusted analysis: infant sex, pregnancy duration, breastfeeding status at month-4, maternal pre-pregnancy BMI, AUC _Glucose_ (OGTT) and gestational weight gain.

### Relation of maternal plasma C-peptide and adiponectin to offspring fat distribution up to year-1

**Figure 1:**
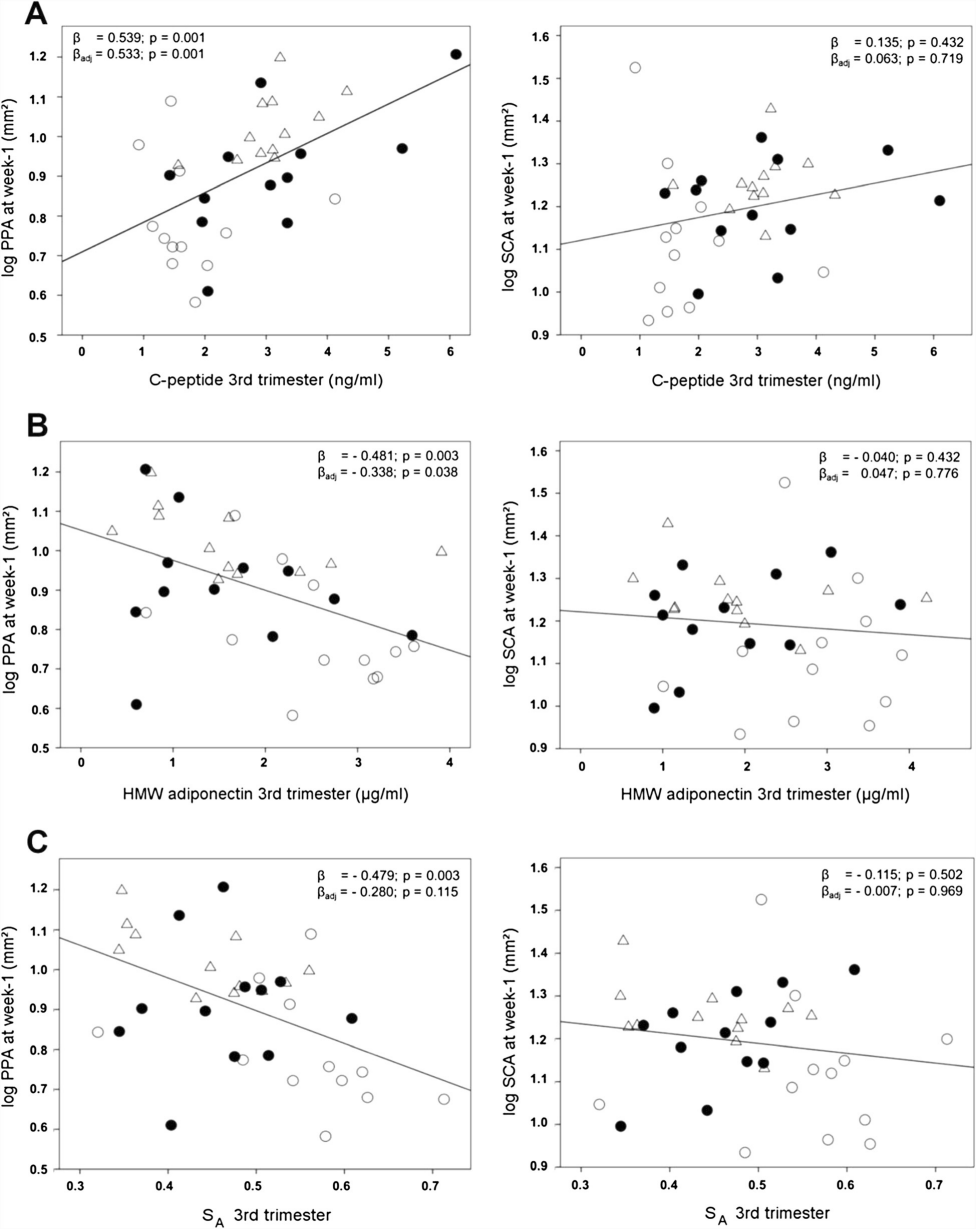
**Regression of PPA and SCA at week-1 on maternal plasma C-peptide and adiponectin levels at 3rd trimester.** Maternal C-peptide **(A)**, HMW adiponectin **(B)** and S_A_**(C)** are presented in relation to neonatal PPA and SCA, respectively. β: unadjusted standardized regression coefficient. β_adj_: standardized regression coefficient adjusted for the variables infant sex, pregnancy duration, maternal pre-pregnancy BMI, AUC _Glucose_ (OGTT) and gestational weight gain. The plotted regression lines indicate the results of the unadjusted analyses. HMW: high molecular weight; Log: logarithmic; PPA: preperitoneal adipose tissue; S_A:_ HMW-total adiponectin ratio; SCA: subcutaneous adipose tissue; open circles: lean group; black circles: obese group; open triangles: obese GDM group.

Linear regression analyses were conducted to determine the relationship of maternal fasted C-peptide, HMW adiponectin levels and S_A_ at 3rd trimester with neonatal PPA at week-1 (Figure 1). For maternal C-peptide, a significantly positive relationship with infant PPA was found in the unadjusted analysis, and C-peptide remained an independent positive predictor after full adjustment for infant sex, pregnancy duration, pre-pregnancy BMI, OGTT-derived AUC _Glucose_ and gestational weight gain. Decreasing levels of maternal HMW adiponectin and S_A_ were significantly related to higher offspring PPA at week-1 in the unadjusted analysis, whereas in the adjusted model, only maternal HMW adiponectin remained as negative determinant. Notably, no significant relationships were found for maternal C-peptide, HMW adiponectin or S_A_ with subcutaneous adipose tissues, namely SCA (Figure 1) and SFT (Additional file 2), at week-1. Indicated maternal parameters were no longer related to PPA at week-6, month-4 and year-1 in the fully adjusted analyses, however we found that maternal HMW adiponectin and S_A_ were significantly positively related to infant SCA only at month-4 (Additional file 2).

## Discussion

Adipose tissue development within the 1st year in offspring born from obese and GDM mothers is poorly investigated. This study is the first reporting comparative longitudinally SFT assessment combined with complementary abdominal ultrasonography of SCA and PPA from birth to year-1 *pp* in offspring from obese mothers with and without GDM and normoglycemic lean mothers.

Our data show that offspring of obese GDM mothers have significantly higher cord insulin levels associated with higher fat mass than offspring of euglycemic lean and obese mothers. The major novel findings were that abdominal SCA and PPA in infants from obese women with GDM were significantly higher at week-1 and SFT was significantly elevated at week-1 and week-6 compared to offspring of lean mothers. Moreover, our analysis revealed that neonatal PPA is highly related to PPA development at year-1. Searching for maternal markers as predictors for offspring adipose tissue growth and obesity risk, we identified a significantly positive relationship for maternal C-peptide levels and a significantly inverse relationship for HMW adiponectin with infant PPA at week-1. However, these associations of maternal factors did not prolong afterwards in the first year of life.

Our data are compatible with the hypothesis proposed by Pedersen et al. [25] indicating that fetal hyperinsulinemia is an adaptive process resulting from elevated maternal glucose levels [6], and that fetal insulin is associated with neonatal fat mass and growth due to the role of insulin in adipogenesis and stimulation of insulin-like-growth factor 1 (IGF-1) production [6,26]. Moreover, the data are in line with two other studies that did not observe fetal hyperinsulinemia in pregnancies of euglycemic obese women [27,28]. In contrast, Catalano et al. [7] reported increased total fat mass and insulin levels in newborns from obese mothers and thereby bringing forward insulin resistance *in utero* without maternal diabetes. However, in the study of Catalano et al. [7], OGTT was performed in the 2nd trimester and a subsequent glucose tolerance challenge in late pregnancy was not performed to exclude GDM in obese women, whereas in our study glucose tolerance was also analyzed in late pregnancy. Our findings on adipose tissue development and distribution within the first year are consistent with data from several studies reporting that GDM offspring overweight at birth diminished within the 1st year *pp*[9,29,30]. Interestingly, these studies found that infant overweight reemerged at the age of 2–3 years. In this context, GDM and reduced maternal insulin sensitivity (IS _OGTT_ index) were reported to be independent predictors for both slower infant weight gain up to month-6 [31] and reduced SFT at year-1 [32]. In contrast, other investigators showed increased body weight and weight/length-ratio in 6-month-old children in relation to pregravid obesity and gestational weight gain or found persistent increased SFT at year-1 in LGA (large for gestational age) offspring born to GDM mothers [33,34].

Apart from maternal variables, cord plasma markers emerged as important indicators for weight and fat mass growth in early childhood; thus, cord insulin levels were inversely related to infant weight gain at year-2 [35]. Moreover, elevated cord blood leptin levels were identified to be related to slower infant weight gain up to month-6 [31], year-2 [36] and year-3 [37]. Altogether, it is tempting to speculate that increased intrauterine and perinatal insulin levels initiated a temporarily prolonged increase in adipose tissues in GDM offspring up to week-6, a postnatal time-period where GDM offspring insulin have returned already to a normal level. Afterwards, during subsequent time up to year-1, weight and adipose tissue growth normalized, because these insulin-mediated effects faded away. However, a long-term impact of maternal and fetal hyperglycemia on later infancy and adolescence obesity risk is still plausible as indicated by epidemiological studies [11,12,38]. In addition, the impact of early postnatal critical periods of programming was highlighted by Péneau et al. [39] showing that high infant BMI at year-1 and early rapid infant weight gain were risk factors for the development and persistence of overweight at the age of 7–9 years.

We found that neonatal PPA at week-1 is an independent predictor for PPA at infant age of 1 year. These data on PPA may mirror intrauterine influences on early adipose tissue development and programming effects with potentially sustained unfavorable effects at later stages in infancy or childhood. This proposed relationship warrants evaluation in future studies with larger cohorts. The importance of investigating specific changes in early adipose tissue distribution as we performed in this study is supported by recent insights into distinct neonatal fat depots assessed by magnetic resonance tomography (MRT) [40,41]. The authors described significantly increased intra-hepatocellular lipids in newborns in relation to pregravid BMI and GDM, but conflicting results have been reported regarding the effect of maternal obesity on abdominal fat in neonates [40,41]. Brumbaugh et al. [40] did not find a difference in intra-abdominal fat per body length in infants of obese mothers with GDM, whereas Modi et al. [41] showed the positive relationship between maternal BMI and abdominal fat in newborns, but did not discriminate between subcutaneous and intra-abdominal fat. Apart from that, PPA was demonstrated to be a good approximation for intra-abdominal fat assessed by computer tomography (CT) [19].

More recently, research interest has started to particularly focus on comprehensively analyzing the distribution and accumulation of distinct fat depots in the body, especially the abdominal fat in longitudinal studies and their relation to metabolic biomarkers. Our data indicate that cord plasma insulin was positively related to neonatal SFT and fat mass that is in line with the results reported by Brunner et al. [35]. Importantly, for the first time, we showed that cord insulin levels emerged as independent predictors for PPA and SCA at week-1 after birth. Interestingly, Tamura et al. [42] have shown that insulin levels are strongly related to PPA and SCA in 9-15-year old children. In addition, Cnop et al. [43] reported that insulin sensitivity was inversely correlated with visceral, but not with subcutaneous fat, whereas leptin levels were stronger related to subcutaneous fat amount in adults. Moreover, increased PPA was recently determined as cardiovascular risk factor in obese children [15] and non-obese adults [16,44]. Considering these studies and our data on cord plasma insulin levels with SCA and PPA in neonates, we speculate that increased neonatal PPA and SCA results from insulin-mediated early metabolic malprogramming which may have the potency to contribute to adverse effects later in life. Therefore, increased PPA at birth might be indicative for both later obesity and cardiovascular risk.

With regard to maternal lipid levels during pregnancy and the recurrently described hypertriglyceridemia in GDM [45,46], we found that total and LDL cholesterol levels were significantly lower in obese women than in the lean group, whereas triglyceride levels were similar in all groups at the 3rd trimester. In addition these maternal differences were not reflected in cord plasma where cholesterol and triglyceride levels were rather alike. Our data on both maternal cholesterol and triglyceride levels for all study groups are consistent with several studies [47-52]. According to Meyer et al. [51] and Scifres et al. [52], obese women exhibit higher levels for triglycerides, total and LDL cholesterol at the beginning of pregnancy than their lean counterparts, but these differences decrease towards term reaching similar or even slightly lower levels in obese women. Regarding the relation between maternal and cord LDL and total cholesterol levels, published data are not consistent, and sometimes even opposing levels between newborn and mother have been described [49,53].

In pregnant women, it is known that HMW and total adiponectin are closely related to glycemia, insulin sensitivity and β-cell function [54]. We found that obese mothers, characterized by hyperinsulinemia and increased HOMA-IR-index, had decreased HMW adiponectin and S_A_ which is in line with recent data from animal and clinical studies reporting that insulin is a negative regulator of adiponectin, preferentially HMW oligomer, secretion [55]. In contrast, in the present study, total and HMW adiponectin levels in cord plasma were not affected by maternal obesity and GDM, as has been described [7,56,57]. Umbilical cord total and HMW adiponectin levels have been associated with neonatal weight, ponderal index and body fat [56,58-60], but similar associations did not emerge in our pilot study (Additional file 3). There was also no significant association between insulin and adiponectin levels in neonates (Additional file 3) indicating that the regulation of fetal adiponectin might be different from adult metabolism [59].

Another important finding of our study was that maternal C-peptide levels were positively and maternal HMW adiponectin levels inversely related to infant PPA at week-1. This strongly suggests that maternal insulin and HMW adiponectin levels are major regulators of maternal metabolism which might impact fetal and newborn PPA growth. Moreover, the observed significant relationships of maternal C-peptide and HMW adiponectin levels with PPA, but not with subcutaneous adipose tissues (SFT and SCA) at week-1 implicate that changes in maternal insulin sensitivity indirectly influences the growth of specific fat compartments during fetal development observed in the neonates.

Although this pilot study was limited by a small participant number and imbalanced offspring sex distribution in the group of obese mothers with GDM as well as the short observational follow-up period, it emphasizes the need to apply ultrasonography as useful method to generate new parameters to characterize longitudinally adipose tissue growth and distribution and their association to blood markers and obesity risk.

## Conclusion

This study shows that maternal pregravid obesity combined with GDM but not maternal obesity alone led to offspring neonatal hyperinsulinemia and increased offspring fat mass until week-6. These findings together with the knowledge from published reports on lean pregnant women with GDM corroborate GDM as the pivotal cause of newborn hyperinsulinemia. However, the observed short adverse impact of intrauterine hyperglycemia on neonatal adipose tissue growth seems to vanish within the first year of life. Importantly, the identification of maternal C-peptide and HMW adiponectin levels as novel potential predictors for distinct PPA growth in newborns might be indicative for obesity risk in later life. Recently, increased PPA was determined as cardiovascular risk factor in obese children and non-obese adults. Therefore, increased PPA at birth might be not only indicative for later obesity risk, but also for cardiovascular risk in adulthood. These aspects should be evaluated in relation to the treatment of pregravid obesity and GDM by dietary and lifestyle intervention studies in larger cohorts, since there is an urgent need for the reduction of offspring obesity and the identification of reliable predictors for offspring obesity risk and its relation to cardiovascular risk factors.

## Abbreviations

BMI: Body mass index; GDM: Gestational diabetes mellitus; HMW: High molecular weight; HbA1c: Hemoglobin A1c; HDL: High-density lipoprotein; HOMA-IR: Homeostasis model of assessment-insulin resistance; ICC: Interclass coefficient; LDL: Low-density lipoprotein; LGA: Large for gestational age; OGTT: Oral glucose tolerance test; pp: Post partum; PPA: Preperitoneal adipose tissue; SA: HMW-total adiponectin ratio; SCA: Subcutaneous adipose tissue; SFT: Sum of the 4 skinfold thickness measurements (biceps + triceps + subscapular + suprailiac); SGA: Small for gestational age.

## Competing interests

All the authors declare the absence of financial interests that may be relevant to the submitted work.

## Authors’ contributions

HH and BLB designed research. KTMS gave scientific advice and clinical support. KU was responsible for data collection and trial management, laboratory analysis and performed the statistical analyses. KP performed inter-observer analysis of ultrasonography data. KG supported statistical analysis. KU and BLB wrote the manuscript. All authors read the manuscript and approved the final version.

## Pre-publication history

The pre-publication history for this paper can be accessed here:

http://www.biomedcentral.com/1471-2393/14/138/prepub

## Supplementary Material

Additional file 1: Figure S1 Inter-observer agreement for the ultrasonographic measurement of preperitoneal and subcutaneous fat assessed by the Bland-Altman plot. From 120 randomly chosen ultrasonographic images, 180 subcutaneous and preperitoneal adipose tissue areas were independently assessed by a second observer. The mean of the differences (bias) was −0.17 mm^2^ and the *Limits of Agreement* were −0.65 mm^2^ (mean - 2 SD) and 0.31 mm^2^ (mean + 2 SD). SD: standard deviation. Click here for file

Additional file 2: Table S1 Regression of infant fat distribution parameter at all ages investigated on plasma maternal C-peptide and adiponectin levels at 3rd trimester. Click here for file

Additional file 3: Table S2 Spearman and partial correlations of cord plasma adiponectin levels with cord plasma insulin and newborn adipose tissue parameters. Click here for file
