# Supplementary material for: Effect of maternal obesity with and without gestational diabetes on offspring subcutaneous and preperitoneal adipose tissue development from birth up to year-1
Source: BMC Pregnancy Childbirth. 2014 Apr 11;14:138. doi: 10.1186/1471-2393-14-138 (PMC4108007; doi:10.1186/1471-2393-14-138)
Supplement: Additional file 1: Figure S1 — Inter-observer agreement for the ultrasonographic measurement of preperitoneal and subcutaneous fat assessed by the Bland-Altman plot. From 120 randomly chosen ultrasonographic images, 180 subcutaneous and preperitoneal adipose tissue areas were independently assessed by a second observer. The mean of the differences (bias) was −0.17 mm2 and the Limits of Agreement were −0.65 mm2 (mean - 2 SD) and 0.31 mm2 (mean + 2 SD). SD: standard deviation. [file 1471-2393-14-138-S1.pdf]

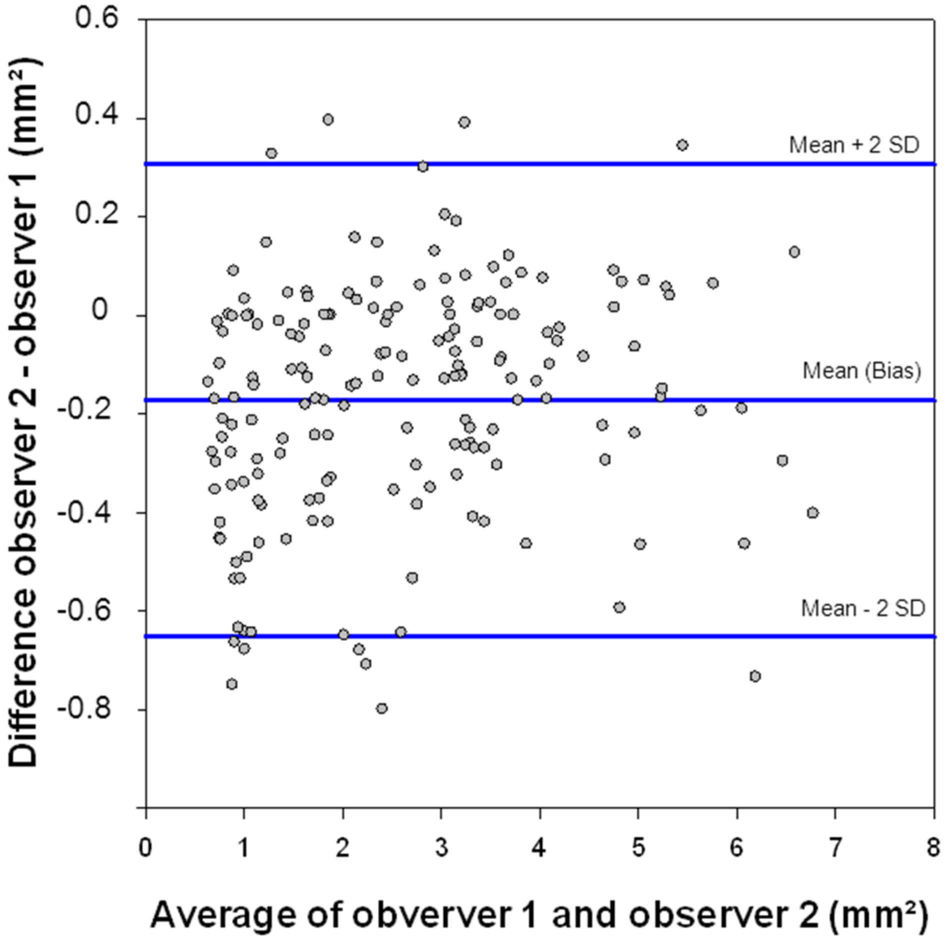


**Figure S1. Inter-observer agreement for the ultrasonographic measurement of preperitoneal and subcutaneous fat assessed by the Bland-Altman plot.** From 120 randomly chosen ultrasonographic images, 180 subcutaneous and preperitoneal adipose tissue areas were independently assessed by a second observer. The mean of the differences (bias) was -0.17 mm$^2$ and the *Limits of Agreement* were -0.65 mm$^2$ (mean - 2 SD) and 0.31 mm$^2$ (mean + 2 SD). SD: standard deviation.
