# Supplementary material for: Effect of maternal obesity with and without gestational diabetes on offspring subcutaneous and preperitoneal adipose tissue development from birth up to year-1
Source: BMC Pregnancy Childbirth. 2014 Apr 11;14:138. doi: 10.1186/1471-2393-14-138 (PMC4108007; doi:10.1186/1471-2393-14-138)
Supplement: Additional file 2: Table S1 — Regression of infant fat distribution parameter at all ages investigated on plasma maternal C-peptide and adiponectin levels at 3rd trimester. [file 1471-2393-14-138-S2.pdf]

Table S1. Regression of infant adipose tissue distribution at all ages on maternal plasma C-peptide and adiponectin levels at 3rd trimester

		Maternal C-peptide					Maternal HMW adiponectin					Maternal S_A				
		Unadjusted analysis		Adjusted analysis			Unadjusted analysis		Adjusted analysis			Unadjusted analysis		Adjusted analysis		
	**N**	**β**	**P-value**	**β**	**P-value**	**Adj. model r^2**	**β**	**P-value**	**β**	**P-value**	**Adj. model r^2**	**β**	**P-value**	**β**	**P-value**	**Adj. model r^2**
**Week-1**																
SFT	43	0.398	0.007	0.325	0.071	0.195; p = 0.026	-0.278	0.068	-0.077	0.638	0.125; p = 0.087	-0.238	0.120	0.031	0.860	0.121; p = 0.093
SCA	36	**0.135**	**0.432**	**0.063**	**0.719**	**0.203; p = 0.046**	**-0.040**	**0.432**	**0.047**	**0.776**	**0.201: p = 0.047**	**-0.115**	**0.502**	**-0.007**	**0.969**	**0.199; p = 0.049**
PPA	36	**0.539**	**0.001**	**0.533**	**0.001**	**0.430; p= 0.001**	**-0.481**	**0.003**	**-0.338**	**0.038**	**0.288; p = 0.012**	**-0.479**	**0.003**	**-0.280**	**0.115**	**0.241; p = 0.026**
**Week-6**																
SFT	40	0.116	0.489	0.256	0.163	0.137; p = 0.110	-0.039	0.812	0.126	0.498	0.075; p = 0.220	-0.157	0.340	0.056	0.283	0.064; p = 0.248
SCA	40	0.073	0.660	0.117	0.515	0.146; p = 0.098	-0.005	0.977	0.146	0.417	0.140; p = 0.101	-0.101	0.537	0.105	0.587	0.130; p = 0.115
PPA	40	0.055	0.739	-0.009	0.960	0.179; p = 0.063	-0.325	0.041	-0.244	0.147	0.260; p = 0.017	-0.326	0.040	-0.181	0.323	0.233; p = 0.026
**Month-4**																
SFT	39	0.009	0.958	0.054	0.785	0.118; p = 0.145	0.105	0.525	0.131	0.479	0.067; p = 0.246	0.054	0.742	0.202	0.295	0.085; p = 0.203
SCA	39	-0.080	0.633	-0.008	0.967	0.050; p = 0.295	0.282	0.081	0.387	0.041	0.088; p = 0.196	0.239	0.143	0.475	0.015	0.138; p = 0.109
PPA	39	0.095	0.565	0.267	0.212	-0.046; p = 0.622	0.014	0.930	0.077	0.694	-0.085; p = 0.780	0.105	0.518	0.148	0.426	0.123; p = 0.718
**Year-1**																
SFT	41	-0.047	0.774	-0.220	0.288	-0.026; p = 0.548	0.169	0.290	0.226	0.234	-0.034; p = 0.583	0.102	0.526	0.208	0.304	-0.045; p = 0.631
SCA	41	0.152	0.348	0.003	0.989	-0.007; p = 0.476	-0.026	0.872	0.109	0.562	-0.029; p = 0.564	-0.090	0.575	0.119	0.552	-0.029; p = 0.561
PPA	41	0.225	0.162	0.202	0.275	0.177; p = 0.061	-0.224	0.159	-0.105	0.539	0.147; p = 0.087	0.084	0.603	-0.137	0.416	0.154; p = 0.079

Adj: adjusted; β: standardized regression coefficient; PPA: preperitoneal adipose tissue; r^2: coefficient of determination; S_A: HMW-total adiponectin ratio; SCA: subcutaneous adipose tissue; SFT: sum of the 4 skinfold thickness measurements (biceps + triceps + subscapular + suprailiac). Variables for adjusted analysis: infant sex, pregnancy duration, respective breastfeeding status, maternal pre-pregnancy BMI, $AUC_{Glucose}$ (OGTT) and gestational weight gain. Values indicated in bold are presented in Figure 1.
