# Supplementary material for: Effect of maternal obesity with and without gestational diabetes on offspring subcutaneous and preperitoneal adipose tissue development from birth up to year-1
Source: BMC Pregnancy Childbirth. 2014 Apr 11;14:138. doi: 10.1186/1471-2393-14-138 (PMC4108007; doi:10.1186/1471-2393-14-138)
Supplement: Additional file 3: Table S2 — Spearman and partial correlations of cord plasma adiponectin levels with cord plasma insulin and newborn adipose tissue parameters. [file 1471-2393-14-138-S3.pdf]

Table S2. Spearman and partial correlations of cord plasma adiponectin with cord plasma insulin and newborn adipose tissue parameters

		Cord plasma HMW adiponectin				Cord plasma total adiponectin				Cord plasma S_A			
	N	r_1	P-value	r_2	P-value	r_1	P-value	r_2	P-value	r_1	P-value	r_2	P-value
Cord plasma insulin	44	0.119	0.442	0.064	0.699	0.121	0.432	0.010	0.951	0.159	0.304	0.150	0.362
Weight	44	-0.101	0.515	-0.047	0.775	-0.086	0.580	-0.032	0.846	-0.146	0.345	-0.232	0.156
Ponderal index	44	-0.150	0.332	-0.134	0.416	-0.143	0.355	-0.129	0.433	-0.143	0.348	-0.274	0.092
SFT	44	0.011	0.941	0.007	0.964	0.016	0.918	0.008	0.964	-0.093	0.546	-0.059	0.721
SCA	36	0.197	0.250	0.173	0.354	0.218	0.201	0.194	0.295	-0.028	0.873	-0.048	0.798
PPA	36	0.140	0.416	0.211	0.234	0.129	0.454	0.237	0.200	-0.038	0.827	0.126	0.500

PPA: preperitoneal adipose tissue; r_1: Spearman correlation coefficient; r_2: partial correlation coefficient adjusted for infant sex, pregnancy duration, maternal pre-pregnancy BMI, $AUC_{Glucose}$ (OGTT) and gestational weight gain; S_A: HMW-total adiponectin ratio; SCA: subcutaneous adipose tissue; SFT: sum of the 4 skinfold thickness measurements (biceps + triceps + subscapular + suprailiac).
